# The DNA Methylation in Neurological Diseases

**DOI:** 10.3390/cells11213439

**Published:** 2022-10-31

**Authors:** Samareh Younesian, Amir-Mohammad Yousefi, Majid Momeny, Seyed H. Ghaffari, Davood Bashash

**Affiliations:** 1 Department of Hematology and Blood Banking, School of Allied Medical Sciences, Shahid Beheshti University of Medical Sciences, Tehran 1971653313, Iran; 2 The Brown Foundation Institute of Molecular Medicine, McGovern Medical School, The University of Texas Health Science Center at Houston, Houston, TX 77030, USA; 3 Hematology, Oncology and Stem Cell Transplantation Research Center, Shariati Hospital, Tehran University of Medical Sciences, Tehran 1411713135, Iran

**Keywords:** DNA methylation, neurological disorders, Huntington’s disease, Alzheimer’s disease, Parkinson’s disease, autism

## Abstract

DNA methylation is critical for the normal development and functioning of the human brain, such as the proliferation and differentiation of neural stem cells, synaptic plasticity, neuronal reparation, learning, and memory. Despite the physical stability of DNA and methylated DNA compared to other epigenetic modifications, some DNA methylation-based biomarkers have translated into clinical practice. Increasing reports indicate a strong association between DNA methylation profiles and various clinical outcomes in neurological diseases, making DNA methylation profiles valuable as novel clinical markers. In this review, we aim to discuss the latest evidence concerning DNA methylation alterations in the development of neurodegenerative, neurodevelopmental, and neuropsychiatric diseases. We also highlighted the relationship of DNA methylation alterations with the disease progression and outcome in many neurological diseases such as Alzheimer’s disease, Parkinson’s disease, amyotrophic lateral sclerosis, frontotemporal dementia, and autism.

## 1. Introduction

DNA methylation is the first discovered epigenetic modification achieved by the enzymatic addition of a methyl group (-CH3) from S-adenosyl methionine to the fifth carbon position of cytosine in a CpG dinucleotide [1]. It is a triphasic process in which DNA methylation groups can be synthesized de novo, maintained, or removed. These processes are mediated by an intricate balance between DNA methyltransferases (writers: DNMT1, DNMT3A, and 3B) and DNA demethylases (erasers: TET1, TET2, and TET3). As well, DNA methylation machinery also needs a group of proteins known as “readers” to translate annotation into functional information [1]. DNA methylation is a normal reversible process used by cells to control gene expression and plays key roles in many biological processes, including embryo development, genomic imprinting, X-chromosome inactivation, repression of transposable elements, and genome stability [2,3]. The DNA methylation patterns established early during embryogenesis tend to be maintained throughout adulthood but may change during embryogenesis and aging [4,5].

In the human brain, DNA methylation and hydroxymethylation processes are critical for the normal development and functioning of the brain, such as the proliferation and differentiation of neural stem cells, synaptic plasticity, neuronal reparation, learning, and memory [6]. Given the environmental influences on the etiology of neurological diseases, many scientists are using epigenome-wide association studies to uncover alterations in DNA methylation associated with these phenotypes [7]. The past decades have seen a growing awareness of the importance of epigenetic mechanisms in the pathogenesis of various neurological diseases. Recent studies have also demonstrated that various DNA methylation alterations in neurological diseases are associated with disease activity, disease progression, and clinical outcome, and may have a prognostic or diagnostic value. The fact that DNA methylation alterations are reversible also makes them a valuable target for therapeutic intervention [8]. In the present review, we aim to discuss the latest evidence concerning DNA methylation alterations in the development of neurological diseases and their relationship with the progression and outcome of disease that support their relevant roles as clinical biomarkers.

## 2. DNA Methylation in Neurodegenerative Diseases

### 2.1. Fragile X Syndrome (FXS)

**Figure 1:**
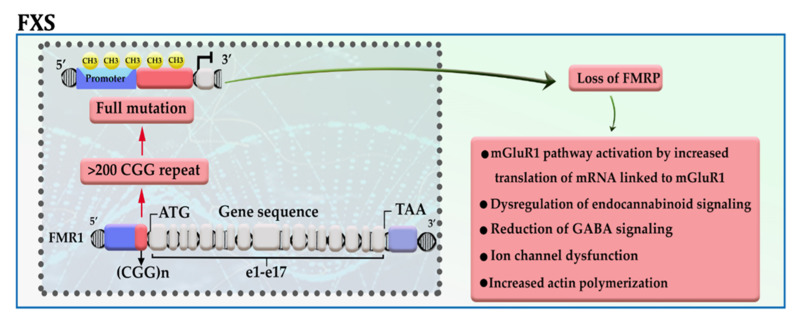
A schematic representation of the selected mechanisms in the pathogenesis of FXS. The presence of more than 200 copies of CGG repeat in the 5′ UTR region of the *FMR1* gene leads to promoter hypermethylation, transcriptional silencing, and loss of FMR protein. mGluRI: group 1 metabotropic glutamate receptors; GABA: gamma-aminobutyric acid.

Fragile X syndrome (FXS), the most common inherited mental retardation, is caused when the expression of the fragile X mental retardation protein (FMRP)—an essential factor for the maintenance of synapses—is downregulated in the brain tissue due to aberrant repeat of CGG trinucleotide in the 5′ UTR of the gene [9,10]. The presence of more than 200 copies of CGG trinucleotide attracts DNA methylation modifiers to the promotor of *FMR1*, thereby disrupting gene transcription. The results of in vitro analysis also confirmed that among different epigenetic modifications, this is DNA methylation has a significant role in the pathogenesis of the disease (Figure 1) [11]. FMRP, an RNA binding protein, has been represented as a molecular brake for the translation of several mRNAs, in particular, mRNAs linked to the activity of metabotropic glutamate receptor group I (mGluRI); hence, it possibly acts as an essential factor for maintaining normal synaptic plasticity. Loss of FMRP expression, in addition to activation of the mGluRI pathway, may lead to dysregulation of endocannabinoid signaling, ion channel dysfunction, reduction of GABA signaling, and increased actin polymerization [10]. Meanwhile, Liu et al. showed that enforcing the expression of *TET1* in neural cells of FXS patients using the dCas9-TET1 CD could reactivate the expression of FMRP by eliminating the methyl groups from the promotor of the FMR1 gene [12].

### 2.2. Huntington’s Disease (HD)

**Figure 2:**
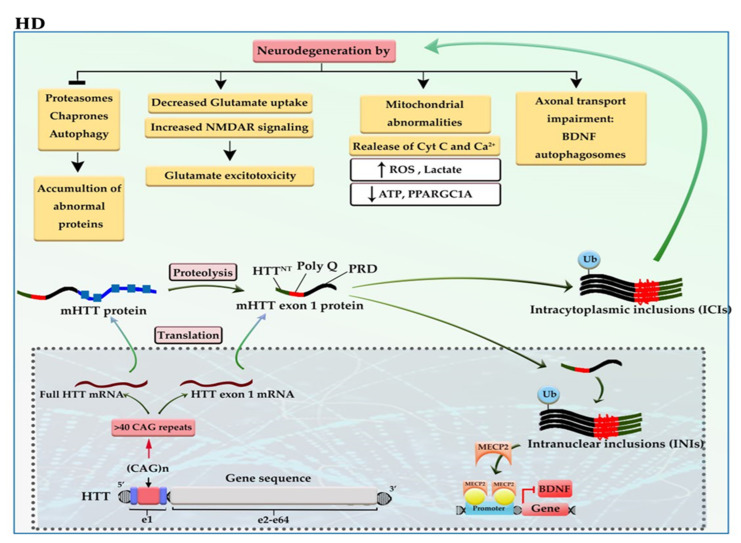
A schematic representation of the selected mechanisms in the pathogenesis of HD. *HTT* gene, located at 4p16, has 64 exons and a CAG trinucleotide repeat expansion in the exon 1 region. The CAG repeats in the coding sequence of mutant *HTT* (mHTT) produce mHTT exon 1 protein. mHTT exon 1 protein enters the nucleus. The gradual aggregation of mHTT exon 1 oligomers leads to the formation of large inclusions in the cytoplasm and nucleus of neural cells. The intracytoplasmic inclusions (ICIs) have various toxic effects on neural cells and can exacerbate the aggregation of mHTT exon 1 protein. Whereas the polyQ tract of intranuclear inclusions (INIs) recruits MECP2 to the promoter of *BDNF*, downregulating the expression of *BDNF*. *BDNF*: brain-derived neurotrophic factor; Cyt-c: cytochrome c; mHTT: mutant huntingtin; NMDAR: N-methyl-D-aspartate receptor; ROS: reactive oxygen species.

The incidence of more than 40 CAG trinucleotide repeats in the huntingtin gene (*HTT*) located on chromosome 4 gives rise to one of the most dreadful neurodegenerative diseases, named Huntington’s disease (HD). The defective HTT protein is toxic to neural cells, especially those that are in subcortical basal ganglia (striatum) [13,14]. The mutant *HTT* (mHTT) produces two transcripts, full-length mHTT mRNA and mHTT exon 1 mRNA (the result of CAG repeat length-dependent aberrant splicing) that are translated into full mHTT protein and mHTT exon 1 protein. Full mHTT protein undergoing proteolysis (by caspases and calpains) is cleaved to generate mHTT exon 1 like protein and other products. mHTT exon 1 protein consists of HTT^NT^ (an N-terminal segment of 17 amino acids), polyQ (a polyglutamine sequence encoded by the CAG repeat), and PRD (a proline-rich domain of 51 amino acids). mHTT exon 1 protein enters the nucleus. The mHTT exon 1 proteins can oligomerize; then, gradual aggregation of oligomers leads to the formation of mHTT fibrils and subsequently large inclusions in the cytoplasm and nucleus of neural cells. Intracytoplasmic inclusions (ICIs) have various toxic effects, including axonal transport impairment, glutamate excitotoxicity, and mitochondrial abnormalities as well as inhibition of proteasomes, chaperones, and autophagy, which can exacerbate the aggregation of mHTT exon 1 protein [13]. While the polyQ tract of intranuclear inclusions (INIs) can alter the interaction with transcription factors and epigenetic modifiers, thereby causing a shift from open to closed chromatin underlies the reduced transcriptional of essential genes, such as *BDNF* and *PPARGC1A* [15]. For example, the polyQ tract of mHTT increases the interaction of mHTT-MECP2 and the recruitment of MECP2 into the *BDNF* promoter, thereby reducing *BDNF* expression (Figure 2) [16].

Upon alteration in methylation patterns such as gain of 5-methylcytosine (5mC), mHTT provides a platform for reducing the expression of genes, which are crucial for neurogenesis, neuronal activity, and survival like *SOX2*, *PAX6*, *NES*, and *BDNF* [17,18]. Moreover, the 5hmC reduction in the striatum and the cortex of transgenic HD mice can inhibit the progression of some essential signaling pathways for neurogenesis, neuronal function, and survival, including Wnt/β-catenin/SOX, NMDAR/calcium/CREB, and GABA type A receptor axes. Global loss of 5-hmC in the cortex may result from the lower expression of *TET1*, whereas global loss of 5-hmC in the striatum may result from downregulation of *TETs* and upregulation of the *MECP2* [19]. It seems that DNA methylation abnormalities may also play a role in reducing the expression of adenosine A2a receptor (ADORA2A) protein, which is one of the earliest events in the pathogenesis of HD. The genome-wide methylation analysis reported the accumulation of methyl groups in exon 1 of the *ADORA2A* gene. OF note, ADORA2A is an essential G-coupled protein for the survival of neural cells in the subcortical basal ganglia [20]. Despite all observations and based on the monogenic nature of the disease, there is a reluctance about accepting the role of DNA methylation in the pathogenesis of HD; however, these conflicts have not affected the early diagnostic value of 5-hmC in HD patients.

### 2.3. Amyotrophic Lateral Sclerosis (ALS) and Frontotemporal Dementia (FTD)

Amyotrophic lateral sclerosis (ALS) and frontotemporal dementia (FTD) are genetically and pathologically heterogeneous disorders to which multiple genetic factors contribute. The excessive expansion of hexanucleotide repeats GGGGCC (G4C2) in the first intron of the chromosome 9 open reading frame 72 (*C9orf72*) gene is the most frequent genetic cause of ALS and FTD [21]. Despite similar origins, the clinical manifestations of ALS and FTD are completely different; while ALS is a disease of motor neuron death [22], FTD is developed as a result of nerve cell loss in the frontal and anterior temporal lobes of the brain [23].

**Figure 3:**
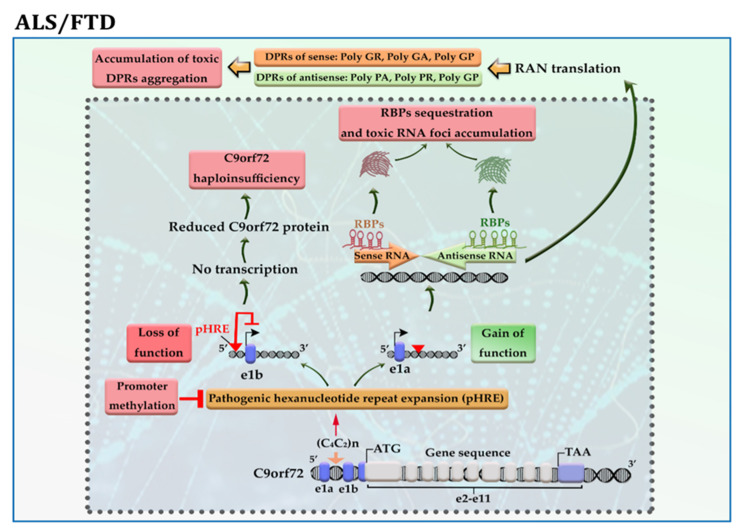
A schematic representation of the selected mechanisms in the pathogenesis of C9-ALS/FTD. Loss of function; the presence of pathogenic hexanucleotide G4C2 repeats expansion (pHRE) within the promoter region inhibits transcription processes and reduces C9orf72 protein levels. Gain of function; the presence of pHRE within intron 1 leads to the sequestration of RNA binding proteins (RBPs) and the accumulation of toxic RNA foci and dipeptide repeat proteins (DRPs). The promoter hypermethylation of the *C9orf72* gene reduces the accumulation of RNA foci and/or DRPs aggregation in the neural cells. Amino acid abbreviations: A: alanine, R: arginine, G: glycine, and P: proline.

The *C9orf72* gene has 11 exons and ≤11 hexanucleotide G4C2 repeats in the vast majority (>95%) of neurologically healthy individuals. This gene produces three transcript variants: Variant 1 encodes the short protein isoform, while Variants 2 and 3 produce the long protein isoform. The presence of pathogenic hexanucleotide G4C2 repeats expansion (pHRE) in intron 1, between non-coding exons 1a and 1b of the gene *C9orf72*, leads to the development of C9-ALS/FTD by loss-of-function and/or toxic gain-of-function mechanisms. When pHRE is located within the promoter region, it can impede transcription processes, leading to a reduction in C9orf72 protein levels and thereby C9orf72 haploinsufficiency. It has been reported that hypermethylation of G4C2 repeat expansion occurs in about 97% of C9-ALS/FTD patients with >50 repeats, which may explain how it triggers the loss of function of the C9orf72 protein [24]. When pHRE is located within intron 1, the pHRE is bidirectionally transcribed into sense and antisense RNAs containing G_4_C_2_ and G_2_C_4_ repeats. The pHRE causes the formation of secondary sense and antisense RNA structures and sequestration of RNA binding proteins (RBPs), leading to the accumulation of toxic RNA foci and impairing RNA processing. In the cytoplasm, sense and antisense RNAs undergo repeat-associated non-ATG (RAN) translation, producing potentially toxic dipeptide repeat proteins (DRPs) from the sense transcript (GA, GR, GP) and the antisense transcript (PA, PR, GP) (Figure 3). Both LoF and toxic GoF mechanisms can change the metabolism pathways and RNA processing. LoF mechanism changes the immune system and microglial function. GoF mechanisms impair proteostasis pathways, mitochondrial function, nucleocytoplasmic transport, transport granule function, and vesicular trafficking. GoF mechanisms can also cause nucleolar dysfunction and affect RNA splicing and transcription, resulting in DNA damage [25].

The promoter hypermethylation and associated silencing of the *C9orf72* gene occur in about 30% of C9-ALS/FTD patients with a favorable prognosis. Further investigations revealed that hypermethylation of the *C9orf72* promoter protects neural cells from cell death by reducing the accumulation of RNA foci and/or DRPs aggregation in the cells, thereby inhibiting their toxic downstream effects [26]. In addition, hypermethylation of *C9orf72* is related to longer survival in patients with C9-FTD [27], while it is related to reduced disease duration before death in patients with C9-ALS [28]. As described above, some phenotypes are likely dependent on the loss of function and others on the gain of function, so hypermethylation could have pleiotropic effects.

Other alterations in DNA methylation reported in both diseases include hypomethylation of the mitochondrial displacement loop (D-loop) region together with increased mitochondrial DNA (mtDNA) copy number in *SOD1*-mutant and sporadic ALS [29]; and hypermethylation of the *GRN* gene in FTD [30]. In ALS, overexpression of *DNMTs* such as *DNMT1* and *DNMT3A*, which participate in the apoptotic death of motor neurons, was observed. Recent evidence also demonstrates an association between overexpression of *DNMTs* and the propagation of mitochondrial-dependent apoptosis in neural cells of spinal cord lesions in ALS mice models [31]. Furthermore, DNA methylation analysis of ALS patients determined that increased DNA methylation (DNAm) age acceleration was associated with shorter survival and earlier age of onset [32].

### 2.4. Alzheimer’s Disease (AD)

**Figure 4:**
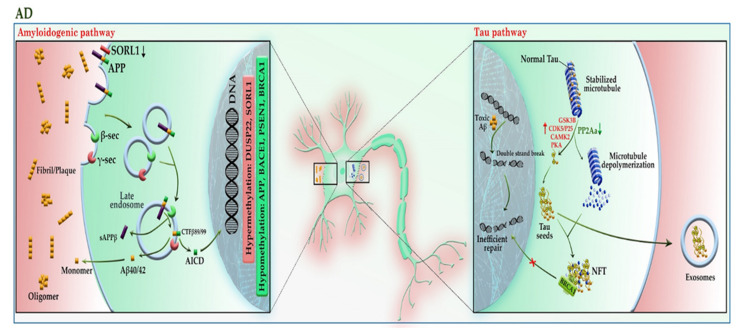
A schematic representation of the selected mechanisms in the pathogenesis of AD. Under physiological conditions, APP is cleaved in the non-amyloidogenic pathway (not shown). In the absence of SORL1 due to epigenetic silencing or mutation, APP is shunted into the late endosomal pathway. In the amyloidogenic pathway, APP enters the late endosome, where it is cleaved by the β-secretase (BACE1), and then by γ-secretase. AICD enters the nucleus and acts as a transcription factor, whereas the Aβ40/42 peptides and sAPPβ are secreted to the extracellular space. An imbalance of Aβ production and its clearance from the brain promotes Aβ aggregation and deposition. The Aβ aggregates can activate the kinases involved in the tau pathway, leading to tau hyperphosphorylation. The aberrant hyperphosphorylation of tau causes p-tau to be separated from microtubules (MTs), leading to MTs depolymerization and axonal degeneration. The disruption of the tau pathway leads to the accumulation of tau aggregates to form oligomers and neurofibrillary tangles (NFTs) within neurons. The p-tau oligomers (tau seeds) can be released into the extracellular space and taken up by unaffected neurons. The tau aggregates sequester BRCA1 protein in the cytoplasm and prevent it from executing its physiological function, leading to the accumulation of DNA damage induced by Aβ. Red and green colors highlighted hypermethylation and hypomethylation, respectively. AICD: amyloid precursor protein (APP) intracellular domain; APP: amyloid precursor protein; BRCA1: breast cancer type 1; CAMK2: calcium/calmodulin-dependent protein kinase II; CDK5: cyclin-dependent kinase 5; CTF-β89/99: β-C-terminal fragment 88/99; DUSP22: dual-specificity phosphatase 22; GSK-3B: glycogen synthase kinase-3B; PKA: protein kinase A; PP2A: protein phosphatase 2; sAAPβ: soluble amyloid precursor protein β; SAM: S-adenosyl methionine; SORL1: sortilin related receptor 1; β-sec: beta-secretase 1; γ-sec: γ-secretase.

Alzheimer’s disease (AD) is the most common form of late-onset neurodegenerative disorder and is characterized by progressive cognitive decline and neuronal death. Evidence demonstrates that mutations in *APP* and *presenilin* genes (*PSEN1* and *PSEN2*) are found in patients with early-onset AD, while *APOE* polymorphism is limited to patients with late-onset AD [33]. In addition to genetic risk factors, analysis of AD brain tissue has taken the veil off the incidence of DNA hypomethylation in low levels of SAM [34]; it not only leads to the reduction of folic acid coupled with an increase in homocysteine in the PB samples of patients [35] but also elevates the expression of genes involved in the amyloid beta (Aβ) pathway, such as *APP*, *PSEN1*, and *BACE1* in the brain [36]. The evidence of DNA hypomethylation in AD patients has also been confirmed in another study, as the number of 5-mC and 5-hmC reduced more significantly in the brain tissue of AD patients compared to healthy individuals [37]. Apart from DNA hypomethylation, the hypermethylation of some genes may also lead to AD. These genes include *ANK1*, *RPL13*, *RHBDF2*, *DUSP22*, and *SORL1* [38,39,40,41]. As shown in Figure 4, the aberrant expression of these genes contributes to the progression of the Aβ and tau pathways. An imbalance in the production and clearance of Aβ and tau aggregates promotes the extracellular accumulation of amyloid plaques and the intracellular aggregation of neurofibrillary tangles (NFT). The forms of Aβ and tau aggregates have various toxic effects on neural cells. The Aβ aggregates lead to activated glial cells induced-neuroinflammation, synaptic toxicity (long-term potentiation (LTP) impairment and long-term depression (LTD) enhancement), mitochondrial dysregulation, and ion channel dysfunction. In addition, Aβ can activate the kinases involved in the tau pathway, leading to tau hyperphosphorylation [33].

Physiologically, tau protein can bind to and stabilize microtubules (MTs). This attachment regulates by the phosphorylation level of tau. The aberrant hyperphosphorylation of tau as a result of hyperactivation of tau kinases or downregulation of tau phosphatases causes phosphorylated-tau (p-tau) to be separated from MTs, leading to MTs depolymerization and axonal degeneration. The hyperactivity of tau kinases may be the result of the effect of Aβ or the epigenetic silencing of their inhibitors, such as DUSP22, which inhibits PKA. In addition, PP2A, a major tau phosphatase, is activated by the addition of a methyl (CH3) group. Under diminished SAM in the brain cells, PP2A inactivation leads to tau hyperphosphorylation. Hyperphosphorylated tau can accumulate to form oligomers, β-sheet-containing structure paired helical filaments (PHFs), and ultimately NFT inside neurons. Apart from axonal degeneration, tau aggregates may impair kinesin-dependent transport, mitochondrial function, and pre- and postsynaptic functions, leading to neuronal cell death. In addition, p-tau oligomers (tau seeds) can be released into the extracellular space via exosomes or directly from the plasma membrane, taken up by unaffected neurons, and tau pathology gradually engages more brain regions as the disease progresses.

Aβ and tau interact in many neuronal compartments, especially at the level of mitochondrial function, leading to impairment of oxidative phosphorylation, mitochondrial transportation, mitophagy, and mitochondrial fission and fusion [33]). In addition to the toxic effects mentioned above, tau aggregates can sequester BRCA1 protein in the cytoplasm and prevent it from executing its physiological function, leading to the accumulation of DNA damage induced by Aβ. It seems that neurons try to cope with this condition by upregulating the expression of the *BRCA1* gene through demethylation of the promoter region [42]. 

Moreover, it has been shown that identifying methylation quantitative trait locus (mQTL) in the approximate *PM20D1* gene is associated with AD development [43]. Recently, many efforts are being accomplished to use methylation patterns as a reliable marker for predicting disease prognosis. For example, greater methylation levels at specific CpG sites of the *BACE1* gene promoter were associated with lower Aβ load and higher tangle density in AD patients with dementia compared to subjects without cognitive impairment (NCI) or mild cognitive impairment (MCI) [36]. *PIN1* hypermethylation can serve as a useful predictive biomarker to distinguish frontotemporal dementia from AD [44]. Moreover, the levels of DNA methylation in the critical genes involved in AD pathogenesis, such as *APP*, *BACE1*, *LRP1*, and *SORL1* may be considered in obese individuals as a sign of AD development in the following years (NCT02868905). These findings together indicate that DNA methylation plays an essential role in AD development even before the onset of the disease.

### 2.5. Parkinson’s Disease (PD)

**Figure 5:**
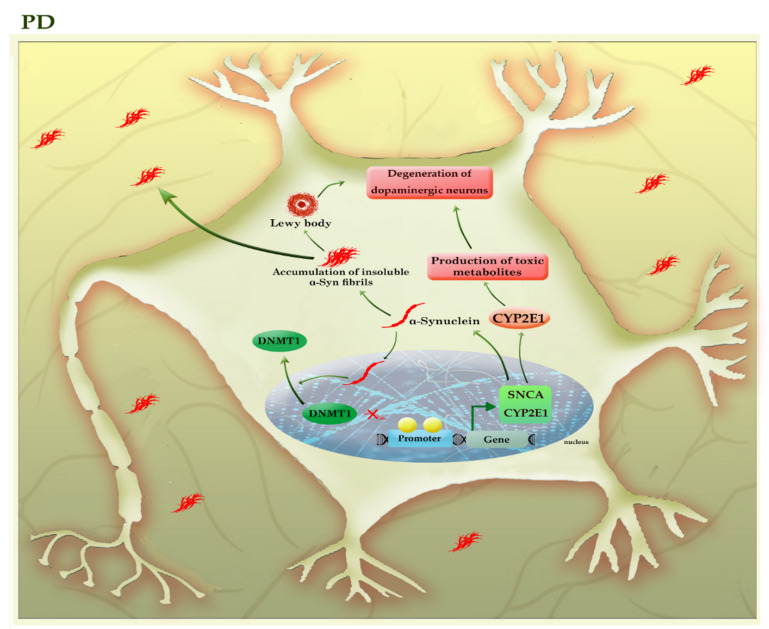
A schematic representation of the selected mechanisms in the pathogenesis of PD. The higher levels of α-synuclein (SNCA) can accumulate to form oligomers, insoluble fibrils, and ultimately “Lewy bodies”, triggering apoptosis within the neural cells. α-synuclein can relocate DNMT1 from the nucleus into the cytoplasm of neural cells and depletes the nuclear reservoir of this DNMT, thereby leading to the hypomethylation and associated with the upregulation of many PD-related genes, including *SNCA* and *CYP2E1*. The increased activity of the *CYP2E1* gene may contribute to the degeneration of dopaminergic neurons by the formation of toxic metabolites. The light green colors highlighted hypomethylated genes. *CYP2E1*: cytochrome P450 2E1; *SNCA* (α-Syn): synuclein alpha.

Parkinson’s disease, the second most common neurodegenerative disorder, is characterized by a widespread intracellular accumulation of the α-synuclein proteins, accompanied by a loss of dopaminergic neurons, both of which occur in the substantia nigra pars compacta of the midbrain. The proper regulation of the α-synuclein (SNCA) is critical for neural health because its higher levels can accumulate to form oligomers, protofibrils, insoluble fibrils, and ultimately “Lewy bodies” in the cytoplasm of neurons, triggering apoptosis within the cells [45]. The existence of mutations or the hypomethylation of CpG islands in intron 1 of the *SNCA* gene enforces the expression of this protein in PD patients [46,47]. Interestingly, α-synuclein can relocate DNMT1 from the nucleus into the cytoplasm of neural cells and depletes the nuclear reservoir of this DNMT, thereby leading to the hypomethylation and associated with the upregulation of many PD-related genes, including *SNCA* and *CYP2E1* (Figure 5).

The impact of DNMT1 on the *SNCA* gene is reversible, as *DNMT1* re-expression in the nucleus could partially diminish the expression of SNCA in the neural cells of transgenic mouse brains [48]. On the other hand, α-synuclein aggregates can be released into the extracellular space and taken up by unaffected neurons, so α-synuclein pathology gradually engages more brain regions as the disease progresses. Toxic effects of various forms of α-synuclein aggregates include activated microglia induced-neuroinflammation and mitochondrial abnormalities, as well as inhibition of the lysosomal autophagy system (LAS) and ubiquitin-proteasome system, exacerbating the aggregation of α-synuclein [45]. Since the disease is silent until after a significant neuronal loss occurs, so identification of early diagnostic biomarkers would be essential to timely diagnosis and treatment. Given the importance of DNA methylation in *SCNA* regulation and based on the fact that epigenetic alterations may occur before the onset of the disease, it has been suggested that the analysis of CpG methylation of the *SNCA* gene in blood samples can use as an early diagnostic method for PD [46].

In addition, promoter hypomethylation and increased activity of the *CYP2E1* gene may contribute to the degeneration of dopaminergic neurons by the formation of toxic metabolites [49]. In addition, it seems that levodopa owes its success in treating PD to its impact on regulating the DNA methylation process [50]. Thus, DNA methylation plays an essential role in the pathogenesis and progression of PD. Table 1 summarizes the results of various studies that highlighted the role of DNA methylation in neurodegenerative diseases.

## 3. DNA Methylation in Neurodevelopmental Disorders

### 3.1. Autism Spectrum Disorders (ASD)

ASD is a group of early-onset neurodevelopmental syndromes that evolve as a combination of both genetic disorders and epigenetic alterations, leading to behavioral and communication defects [54]. Among different mechanisms, it seems that epigenetic modulation, in particular alterations in the methylation pattern of the genes, may hold a respectable share in the development of the disease. The dysregulation of DNA methylation in ASD exists on multiple levels: de novo mutations, epigenetic silencing, increased expression in genes encoding epigenetic machinery, abnormally methylated genes, and genome-wide hypo- or hypermethylation. Sperm analysis of a wide range of fathers declared that the amount of DNA methylation alteration related to ASD was remarkably higher in older fathers than their younger counterparts. Studies indeed succeeded in identifying more than 805 differentially methylated regions (DMRs) in sperm that may increase the susceptibility of offspring to ASD [55]. Despite the difficulties in sample collection and analysis, so far, serval mutations in genes encoding methylation writers, readers, and erasers such as *DNMT3A*, *TET2*, *MECP2*, and *MBD5* have been identified in patients with ASD [56,57,58,59]. Congenital epigenetic diseases, such as Angelman syndrome (AS), Rett syndrome (RTT), MECP2 duplication syndrome (MDS), TET3 deficiency, and Fragile X syndrome (FXS), account for less than 10% of cases of ASD and intellectual disability. *MECP2* is also known to be hypermethylated and downregulated in the brains of ASD patients. In mouse models, induction of methylation in the *MECP2* promoter by using the dCas9-based DNA methylation-editing method reduces the expression of *MECP2*, inducing autism-like behaviors [60]. In addition, increased MECP2 interaction with *RELN* and *GAD1* gene promoters triggers the reduction of Reelin and GAD67 expression in the brain regions of patients with ASD [61]. Postmortem human brain studies from ASD patients have highlighted the role of the dysregulated DNA methylation profiles, which affect genes involved in neural, GABAergic, and immune processes [62,63,64,65].

**Figure 6:**
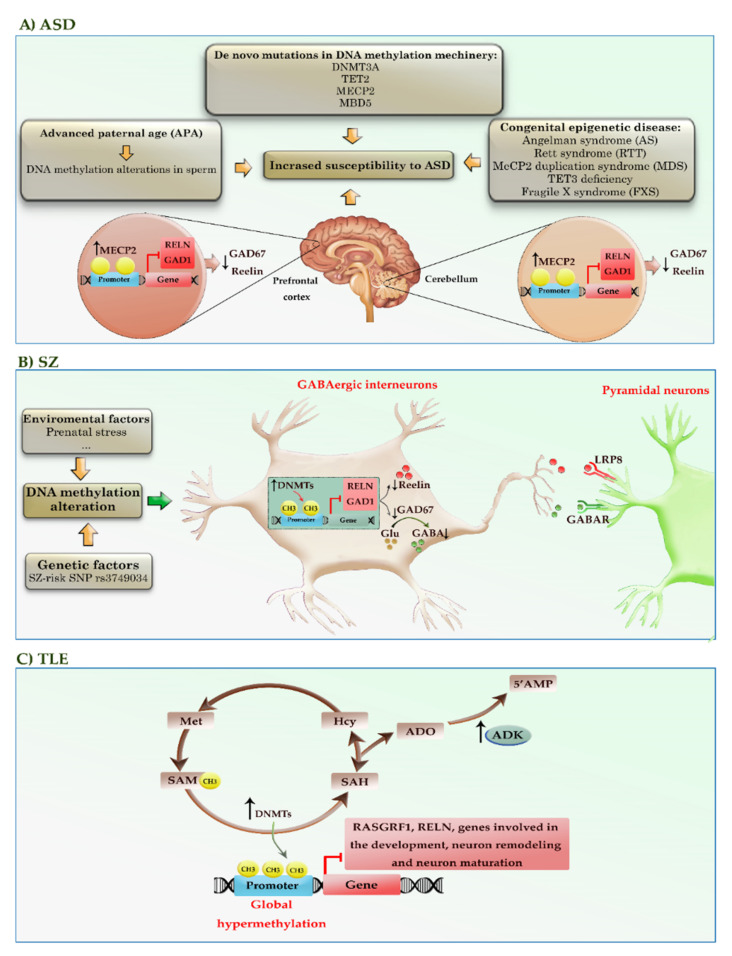
A schematic representation of the DNA methylation alterations in the pathogenesis of neurodevelopmental and neuropsychiatric diseases, such as ASD, SZ, and TLE. (**A**) Advanced paternal age (APA), congenital epigenetic diseases, and de novo mutations in genes encoding DNA methylation machinery may increase the susceptibility of offspring to ASD. In addition, increased MECP2 interaction with *RELN* and *GAD1* gene promoters triggers the reduction of Reelin and GAD67 expression in the brain regions of patients with ASD. (**B**) Environmental factors and certain genetic factors (e.g., rs3749034, an SZ-risk SNP) may cause aberrant methylation and associated silencing of genes, such as *RELN* and *GAD1*. In addition, the increased binding of MECP2 into the promoter of these genes can reduce the expression of Reelin and GAD67. Decreased GAD67 leads to reduced GABA release at synapses and compensatory regulation of postsynaptic GABA_A_ receptors located in pyramidal neurons. (**C**) The alterations in the transmethylation pathway and adenosinergic signaling can lead to global hypermethylation of the genome in epileptogenic areas of the brain. Red colors highlighted hypermethylated genes. ADO: adenosine; AHCY: adenosylhomocysteinase; AMP: adenosine monophosphate; ADK: adenosine kinase; GAD1, 67: glutamic acid decarboxylase1, 67; GABA: gamma-aminobutyric acid; Hcy: homocysteine; RELN: Reelin; *RASGRF1*: Ras protein-specific guanine nucleotide releasing factor 1; SAM: S-adenosyl methionine; SAH: S-adenosyl homocysteine; SNP: single-nucleotide polymorphism; Met: methionine.

To date, limited studies have identified the methylation status of genes as potentially reliable diagnostic markers in ASD patients. For example, the methylation levels of the *BDNF* gene can serve as a useful diagnostic marker in peripheral blood samples (PB) of children with ASD [66]. In addition, the hypermethylation of a CpG site (cg20793532) in the *PPP2R2C* promoter can potentially use as a blood biomarker for identifying adult patients with high-functioning ASD [67]. Taken together, the abstract of these observations not only highlighted the importance of DNA methylation in the formation of ASD but also shed light on the possibility of their application in the early diagnosis of this neurodevelopmental disorder. Table 2 summarizes the results of various studies that highlighted the role of DNA methylation in ASD. Figure 6A provides a schematic representation of the role of DNA methylation alterations in the pathogenesis of ASD.

### 3.2. Rett Syndrome

Rett syndrome (RTT) is a severe neurological disorder with defects in intellectual and lingual abilities as well as motor impairments in women that mainly result from mutations in *MECP2* [72]. *MECP2* gene is located on the X chromosome and encodes a DNA methylation reader protein, which is vital for the normal development and the function of neurons [73]. Given the X-linked property of *MECP2*, RTT has been reported in rare cases of males with a similar phenotype as *MECP2* mutation in men, which is lethal [74]. Recent studies have indicated that *MECP2* plays an essential role at different developmental stages, including prenatal neurogenesis, pre- and post-natal development of synaptic connections and functions, and experience-dependent synaptic maturation and plasticity. *MECP2* is also vital for the acting of various brain circuits by maintaining a balance between synaptic excitation and inhibition [72].

According to the revised clinical criteria [75], the patients are clinically classified into two main groups of RTT: typical/classic and atypical/variant that differ by their symptoms or by the specific gene mutation. The vast majority of patients who strictly meet clinical criteria for typical/classic RTT (over 90% of the case) have *MECP2* mutations. Notably, eight ‘hotspot’ mutations have been identified in more than 60% of documented cases (encoding the amino acid substitutions R106W, R133C, T158M, R168X, R255X, R270X, R294X, and R306C) [76]. So far, several variants of atypical RTT have been reported, such as the preserved speech variant (Zappella variant; is the most common variant, and mutations in *MECP2* are seen in the majority of cases), the early seizure variant (Hanefeld variant; mutations in *CDKL5* seen in the majority of cases), and the congenital variant (Rolando variant; mutations in *FOXG1* seen in the majority of cases), etc. [75].

MECP2 protein contains an N-terminal domain (NTD), an MBD, an intervening domain (ID), a TRD, and a C-terminal domain (CTD), as well as three AT-hook motifs. These domains contribute to a dual regulatory function of MECP2 in the gene expression as a repressor or activator of transcription. In normal conditions, MECP2 binds to the methylated CpG (mCG) dinucleotides on the promotor of some specific genes via its MBD and recruits the SIN3A/HDAC or NCoR/SMRT corepressor complexes via its TRD to repress the expression of the genes involved in synaptic function in the brain cells, thereby regulating excitatory synaptic strength by controlling the number of glutamatergic synapses [77,78]. In addition to binding to mCG, MECP2 binds to methylated CA (mCA) sites [79], methylated CAC (mCAC) trinucleotides [80], and 5-hydroxymethylcytosine (5hmC) [81]. The binding of MECP2 to mCA within the gene body of long genes results in the repression of long genes; therefore, the long genes aberrantly express in RTT [79]. MECP2 acts as a 5hmC binding protein in the brain and facilitates gene transcription in neural cell types; however, in RTT syndrome, an R133C mutation of *MECP2* impairs the 5-hmC binding [81]. Therefore, the specific mutations in *MECP2* can differentially affect MECP2 functions and induce a subset of RTT phenotypes with different severities.

## 4. DNA Methylation in Neuropsychiatric Diseases

### 4.1. Schizophrenia (SZ)

Brain analysis of patients with schizophrenia (SZ) revealed a reduction in the number of neural progenitor cells (NPCs) in the postmortem brains [82]. Since the incidence of SZ, like other neuropsychiatric disorders, is accelerated by environmental factors, it is postulated that the DNA methylation machinery may have a role in the pathogenesis of the disease. In agreement, increased expression of *DNMT1* and *DNMT3A* has been reported in the cortical interneurons of SZ patients [83,84]. Environmental factors such as prenatal stress elevate the expression levels of *DNMT1* and *DNMT3A* in GABAergic interneurons; it induces aberrant promoter methylation and associates with the silencing of genes, such as *RELN* and *GAD1* [85]. Certain genetic factors (e.g., rs3749034, an SZ-risk single-nucleotide polymorphism (SNP)) may also cause aberrant methylation in the regulatory region of the *GAD1* gene [86]. In addition to aberrant methylation, the *RELN* and *GAD1* gene promoters show increased binding of MECP2 [85]. These changes in DNA methylation machinery can reduce the expression of Reelin and GAD67, encoding by *RELN* and *GAD1* genes, respectively (Figure 6B). The extracellular matrix protein Reelin has a crucial role in neuronal migration and the extension of axons and dendrites in the developing cortex, as well as the release of neurotransmitters and synaptic plasticity in the adult brain [87]. A decrease in Reelin may result in reduced dendritic spine densities, GRIN1 (also known as NMDA) receptor dysfunction, and LTP impairment in SZ patients [88]. GAD67 catalyzes the conversion of glutamate to GABA, which is the principal inhibitory neurotransmitter. Decreased GAD67 leads to reduced GABA release at synapses and compensatory regulation of postsynaptic GABA_A_ receptors located in pyramidal neurons. This dysfunction of GABA may impair the synchronization and inhibition of excitatory pyramidal neurons, leading to behavioral and cognitive deficits in SZ patients [89]. Additionally, the analysis of 166 human fetal brain samples succeeded in finding several SZ-associated mQTLs at DNA sequence motifs of chromatin looping protein CTCF, a regulatory protein that represses the expression of several genes [90]. Overall, although evidence supports the involvement of DNA methylation in the pathophysiology of SZ, further analysis is required to specifically relate psychological/behavioral changes of SZ to the epigenetic alterations.

### 4.2. Epilepsy

When Kobow et al. used methylation sequencing analysis in epileptic rats, they took a veil off the involvement of one important regulatory pathway in the incidence of epilepsy. They reported that not only is global hypermethylation a common event in epilepsy, but also it was influenced by the diet. It seems that a ketogenic diet could reduce the number of methylation sites in epileptic rats and ameliorate clinical symptoms [91]. As shown in Figure 6C, alterations in the transmethylation pathway and adenosinergic signaling can lead to global hypermethylation of the genome in epileptogenic areas of the brain. In epilepsy patients, the elevated expression of *DNMT1* and *DNMT3A* adds more methyl groups from SAM to cytosine residues, which may suppress the expression of essential genes such as *RASGRF1*, *RELN*, and genes involved in neuronal development, regeneration, and neuronal maturation [92,93,94,95]. In the process, SAM is converted to S-adenosyl homocysteine (SAH), which is subsequently cleaved to adenosine (ADO) and homocysteine (Hcy) by adenosylhomocysteinase (AHCY). ADO is converted to adenosine monophosphate (AMP) by the enzyme adenosine kinase (ADK), whereas Hcy is converted to methionine (Met) and then back to SAM. The direction of the transmethylation pathway depends on the continuous removal of the obligatory end products ADO and Hcy. In epileptogenic conditions, astrogliosis leads to increased expression of ADK, reducing inhibitory ADO around synapses. ADO deficiency shifts the transmethylation pathway in favor of converting SAM to SAH, thereby increasing DNA methylation as a prerequisite for progressive epilepsy. This finding was so striking that adenosine augmentation therapy, which can diminish the amount of DNA methylation in the brain, has been integrated into the treatment protocol of epilepsy to prevent seizures [96].

Moreover, DNA hypermethylation in epileptic patients can affect the expression of several long non-coding RNAs (lncRNAs), which are involved in the regulation of ion/gated channel activity, GABA receptor activity, and synaptic transmission. Analyzing the blood samples of the patients also showed that about 85% of miRNAs are differentially methylated in epilepsy. Bioinformatic analyses suggested that most methylated miRNAs participate in axonal guidance, neuronal projection development, neuronal differentiation, and protein kinase activity [97]. Table 3 summarizes the results of various studies that highlighted the role of DNA methylation in neuropsychiatric diseases.

## 5. Conclusions and Future Perspectives

Despite numerous studies trying to elucidate the importance of DNA methylation machinery in the development of neurological diseases, the translation of this knowledge into clinical practice is in a nascent stage. This may be due to difficulty in accessing the target tissue, and in most cases, biomarkers can only be measured postmortem (that is, by brain biopsy). On the other hand, the tissue- or, better to say, a cell-specific feature of DNA methylation makes analysis difficult, as there is always a need for primary disease-affected tissues [105,106]. To tackle this obstacle, many claims to use blood samples as a surrogate for affected brain tissue; however, there is a possibility that disease-associated epigenetic alteration may change in surrogate tissues [100,107,108]. Alternatively, disease-affected tissue might have varying DNA methylation patterns, which may not be detectable in surrogate samples [109]. Furthermore, most cell populations contain multiple cell types that may have different DNA methylation patterns. Despite the challenges mentioned above, recent evidence has shown that various DNA methylation alterations in neurological diseases are associated with disease activity, disease progression, and clinical outcome and may have a prognostic or diagnostic value. Studying DNA methylation profiles using cell-free circulating DNA blood (cfDNA) tests [110] can serve as diagnostic and prognostic biomarkers in neurological diseases. The fact that DNA methylation alterations are reversible also makes them a valuable target for therapeutic intervention. In conclusion, identifying DNA methylation-based biomarkers may propose a new patient-tailored therapeutic approach for neurological diseases.

## Figures and Tables

**Table 1:** Potential DNA methylation markers in neurodegenerative diseases.

	Cell/Tissue Type	Main Findings	Ref.
Neurodegenerative Diseases			
**HD**	Brain tissues from HD patients and HCs	Increased 5mC, together with reduced 5hmC levels, were detected in the 5’UTR region of the *ADORA2A* gene in the putamen of HD patients compared to HCs.	[20]
**ALS**	Postmortem brain and spinal cord samples from sporadic ALS and HCs	*DNMT1* and *DNMT3A* were upregulated in the motor cortex and spinal cord motor neurons of patients with sporadic ALS compared to HCs. 5-mC was detected in the motor cortex of ALS but not in HCs.	[31]
	Postmortem frozen spinal cord samples and WB from sporadic ALS and HCs	Global 5-mC and 5-hmC were increased in the spinal cord, but not WB of patients with sporadic ALS compared to HCs.	[51]
	DNA blood from patients with ALS and HCs	*C9orf72* promoter hypermethylation was associated with reduced disease duration before death in patients with C9-ALS.	[28]
	PBMCs from sporadic ALS patients, HCs, and familial ALS patients with SOD1- or C9orf72-mutant	The hypomethylation of the mitochondrial D-loop region, together with increased mtDNA copy number, could represent compensatory mechanisms to counteract mitochondrial impairment in *SOD1*-mutant and sporadic ALS patients.	[29]
	Blood and CNS tissues from sporadic ALS patients	Blood/CNS-based DNAm-age acceleration may be used as a marker to predict the age of onset and survival in ALS patients.	[32]
**ALS, FTD**	Brain or blood samples from C9-ALS/FTD patients and HCs	The hypermethylation of G4C2 repeat expansion occurs in about 97% of C9-ALS/FTD patients with >50 repeats. It was found in both blood and brain tissues for the same individual, suggesting its potential use as a biomarker.	[24]
**FTD**	Brain or blood samples from C9-FTD patients and non-carrier family members	*C9orf72* promoter hypermethylation was associated with longer survival in patients with C9-FTD.	[27]
	Brain samples from HCs, FTD, AD, and PD patients	Promoter hypermethylation and associated silencing of the *GRN* gene were detected in patients with FTD compared to HCs, AD, and PD samples.	[30]
	PBMCs from FTD patients and HCs	Promoter hypermethylation associated with a reduced mRNA expression of the *GRN* gene was found in PB of patients with FTD compared to HCs.	[52]
**AD**	Postmortem human brains from AD patients, donors with NCI and MCI	The greater methylation levels at specific CpG sites of the *BACE1* gene promoter were associated with higher tangle density and lower β-amyloid load among persons with AD dementia than subjects with NCI or MCI.	[36]
	Neurons of postmortem brain samples from AD patients and HCs	Promoter hypomethylation and increased mRNA and protein expression of *BRCA1* was detected in the neurons of hippocampal and entorhinal cortex from AD patients compared to HCs.	[42]
	PFC neurons of Postmortem human brains from AD patients and HCs	Hypomethylation of the enhancers in the *DSCAML1* gene, which targets the *BACE1* promoter, caused the overexpression of *BACE1* in AD patients; and was correlated with an increase in Aβ plaques, NFTs, and cognitive decline.	[53]
	PBMCs from AD patients, FTD donors, and HCs	*PIN1* hypermethylation can serve as a useful predictive biomarker to distinguish AD from FTD.	[44]
	PBMCs from HCs, young obese females, or AD donors	Methylation levels of genes involved in AD pathogenesis, such as *APP*, *BACE1*, *LRP1*, and *SORL1*, can serve as prognostic biomarkers in obese individuals.	NCT02868905
**PD**	PBMCs from sporadic PD patients and HCs	The methylation status of *SNCA* intron-1 can be used as an early diagnostic marker for PD.	[46]
	Postmortem human brains from PD patients and HCs	Promoter hypomethylation and the increased activity of *CYP2E1* may contribute to the degeneration of dopaminergic neurons by the formation of toxic metabolites.	[49]

AD: Alzheimer’s disease; Aβ: amyloid β; ALS: amyotrophic lateral sclerosis; *APP*: amyloid beta precursor protein; *ADORA2A*: adenosine A2a receptor; *BACE1*: beta-site amyloid precursor protein cleaving enzyme 1; *BRCA1*: breast cancer type 1 susceptibility protein; CB: cerebellum; C9-ALS: C9orf72-associated ALS; CNS: central nervous system; C9-ALS/FTD: C9orf72-associated ALS/FTD; C9-FTD: C9orf72-associated FTD; *CYP2E1*: cytochrome P450 family 2 subfamily E member 1; *C9orf72*: chromosome 9 open reading frame 72; *DNMT*: DNA (cytosine-5)-methyltransferase; D-loop: displacement loop; DNAm: DNA methylation; *DSCAML1*: Down syndrome cell adhesion molecules like 1; FTD: frontotemporal dementia; *GRN*: granulin precursor; HD: Huntington’s disease; HCs: healthy controls; 5-hmC: 5-hydroxymethylcytosine; *LRP1*: low-density lipoprotein receptor-related protein 1; mtDNA: mitochondrial DNA; MCI: mild cognitive impairment; 5-mC: 5-methylcytosine; NFTs: neurofibrillary tangles; NCI: no cognitive impairment; PD: Parkinson’s disease; PFC: prefrontal cortex; PB: peripheral blood; PBMNs: peripheral blood mononuclear cells; *PIN1*: peptidyl-prolyl cis-trans isomerase NIMA-interacting 1; SOD1: superoxide dismutase 1; *SORL1*: sortilin related receptor 1; *SNCA*: synuclein alpha; WB: whole blood.

**Table 2:** Potential DNA methylation markers in autism spectrum disorders.

Cell/Tissue Type	Main Findings	Ref.
ASD		
Postmortem brain tissues from ASD patients and HCs	Increased MECP2 interaction with *RELN* and *GAD1* gene promoters triggers the reduction of Reelin and GAD67 expression in the CB and FC of patients with ASD compared to HCs.	[61]
Postmortem brain tissues from ASD patients and HCs	Hypomethylation and overexpression of immune-related genes (such as *C1Q*, *C3*, *ITGB2*, and *TNF-α*) were observed in the PFC of ASD compared to HCs.	[62]
Postmortem tissues from ASD patients and HCs	Hypomethylation of CpG sites in the promoters of immune genes leads to an upregulated immune process in the convergent subtype.	[63]
Frozen brain samples from ASD and HCs	A total of 58 ASD-associated DMRs were enriched for genomic regions of neuronal, GABAergic, and immune system genes.	[64]
Cord, blood, and brain tissues from ASD and HCs	ASD-associated meQTLs across the genome were enriched for immune-related pathways in the cord, blood, and brain tissues of children with ASD.	[65]
PBMCs from children with ASD and HCs	Methylation and expression levels of *BDNF* in blood samples from children with ASD can use as a diagnostic biomarker.	[66]
PB from adults with high-functioning ASD and HCs	Hypermethylation of a CpG site (cg20793532) in the *PPP2R2C* promoter can serve as a blood biomarker for identifying adult patients with high-functioning ASD.	[67]
Blood DNA from male ASD patients and HCs	Most of the 700 DMCpGs (587; 83.9%) in ASD cases showed relative hypomethylation compared to HCs. Hypomethylation and overexpression of *ERMN* contribute to ASD susceptibility and can be altered by both rare SNPs at the CG position and mutations.	[68]
WB samples from ASD-discordant MZ twins, ASD-concordant MZ twins, and a set of pairs of sporadic case-control	A total of 2,397 DAGs were associated with neurotrophin signaling pathway in ASD-discordant MZ twins. The aberrant methylation of *SH2B1* was identified in the ASD-discordant, ASD-concordant MZ twins, and sporadic cases compared to controls.	[69]
Lymphoblastoid cells from idiopathic ASD and unaffected sex-matched siblings	DAGs were associated with synaptogenesis, semaphorin, and mTOR pathways in idiopathic ASD compared to unaffected sex-matched siblings.	[70]
Placenta samples stored from children later diagnosed with ASD compared to typically developing controls	A total of 400 DMRs can distinguish placentas stored from children later diagnosed with ASD relative to typically developing controls. Methylation levels of two DMRs, mapping on *CYP2E1* and *IRS2*, can serve as a useful predictive biomarker for ASD risk in placenta samples.	[71]

ASD: autism spectrum disorder; *BDNF*: brain-derived neurotrophic factor; CB: cerebellum; *C1Q*: complement component 1q; *C3*: complement component 3; *CYP2E1*: cytochrome P450 family 2 subfamily E member 1; DMRs: differentially methylated regions; DMCpGs: differentially methylated CpGs; DAGs: DMR-associated genes; *DNMT*: DNA (cytosine-5)-methyltransferase; *ERMN*: ermin; FC: frontal cortex; *GAD1*: glutamate decarboxylase 1; GAD67: glutamate decarboxylase 67; HCs: healthy controls; *ITGB2*: integrin subunit beta 2; *IRS*: insulin receptor substrate; MZ: monozygotic; meQTLs: methylation quantitative trait loci; mTOR: mechanistic target of rapamycin; PFC: prefrontal cortex; PB: peripheral blood; PBMNs: peripheral blood mononuclear cells; *PPP2R2C*: protein phosphatase 2 regulatory subunit b gamma; PMS: polymethylation score; *RELN* Reelin; *SH2B1*: SH2B adaptor protein 1; *TNF-α*: tumor necrosis factor-α; WB: whole blood.

**Table 3:** Potential DNA methylation markers in neuropsychiatric diseases.

	Cell/Tissue Type	Main Findings	Ref.
Neuropsychiatric Diseases			
**SZ**	Human Brain tissue from SZ patients and HCs	Increased *DNMT1* expression and subsequently elevated DNA methylation levels were detected in SZ patients compared to HCs.	[83]
	Brain tissue and PBL from SZ patients and HCs	The mRNA expression of *DNMT1* and *DNMT3A* was increased in both brain tissue and PBL of SZ patients compared to HCs.	[84]
	PBL from SZ patients and HCs	The mRNA expression of *DNMT1*, *TET1*, *GCortR*, and *BDNF* was increased in PBL of SZ patients compared to HCs.	[98]
	Human fetal and adult brain tissue	>16,000 fetal brain mQTLs were identified. Fetal brain-specific mQTLs were enriched among SZ-associated SNPs identified in a recent study.	[90]
	Blood and brain tissue from SZ-discordant MZ twins, SZ patients, HCs	25 DMPs associated with SZ (*p*-value < 10^−7^). The seven meQTLs were enriched for schizophrenia risk variants in both brain and blood samples.	[99]
	Genome-wide DNA methylation data from WB samples and postmortem DLPFC samples from SZ patients and HCs	Blood PMS signature can distinguish SZ patients from HCs and several other major neuropsychiatric disorders, enriched for methylation differences detected in DLPFC postmortem samples and was correlated with altered functional DLPFC-HC coupling during working memory and biological pathways with synaptic function.	[100]
	Postmortem PFC brain tissue from SZ patients and HCs (the results from three independent studies)	The seven DMRs identified in near *CERS3*, *DPPA5*, *PRDM9*, *DDX43*, *REC8*, *LY6G5C* genes, and a region on chromosome 10 across all three PFC brain data sets may play an important role in the pathogenesis and progression of SZ patients.	[101]
	Genome-wide DNA methylation data of WB samples from SZ patients and HCs	Accelerations in 3 mortality clocks in SZ may result from smoking and 6 age-associated proteins. 2 mitotic clocks were decelerated in SZ related to NK and CD8^+^ T cells and may be a biological basis for reduced cancer risk. Chronological age clocks were decelerated in patients treated with clozapine.	[102]
	Postmortem brain tissue of SZ patients and HCs	The methylation levels of two CpG sites within the 5′ UTR of *GAD1* were significantly associated with SZ-risk SNP rs3749034 and GAD25 expression in DLPFC. The expression of full-length *GAD1* transcript encoding GAD67 was significantly higher in DLPFC of SZ patients who died through suicide.	[86]
**TLE**	Brain tissue of TLE patients and HCs	The expression of *DNMT1* and *DNMT3A* was increased in TLE patients relative to HCs, especially in NeuN^+^ neurons, but not GFAP^+^ astrocytes.	[92]
	Postmortem brain tissue of TLE patients with and without FS and HCs	The levels of DNMT3A1 and DNMT3A2 isoforms were decreased in the hippocampus of TLE patients with FS relative to HCs and other TLE groups. Increased levels of DNMT1, DNMT3A1, and global DNA methylation were found in the neocortex of all TLE patients compared to HCs.	[103]
	Postmortem hippocampus from TLE patients and HCs	81.5% of 146 differentially methylated protein-coding gene promoters were hypermethylated in TLE patients relative to HCs, and these genes are related to development, neuron remodeling, and neuron maturation. Four differentially methylated lncRNAs and 13 methylation-sensitive miRNAs were identified. miR-876-3p was associated with WG1 hippocampal sclerosis.	[95]
	Postmortem hippocampus from TLE patients with and without GCD, and HCs	*RELN* promoter methylation was higher in TLE patients than in HCs. Increased methylation of the *RELN* promoter was associated with GCD among TLE patients.	[94]
	PB DNAs of TLE patients and HCs	85% and 87% of differentially methylated miRNA and lncRNA promoters were hypermethylated in TLE patients compared to HCs. The aberrantly methylated miRNAs and lncRNAs were correlated to drug metabolism, ion channel activity, MAPK- and neurotrophin signaling pathways.	[97]
	WB of MLTE patients and HCs	216 DAGs, with 52 sites involved in hypo- and 164 sites hypermethylation, related to pathways involved in drug metabolism, anion binding, growth regulation, oxidoreductase activity, and skeletal development, with the most distinct ones including *CYP3A43*, *CYP3A4*, *CYP2C9*, *CLCA4*, *CLCN6*, and *SLC34A2.*	[104]

*BDNF*: brain-derived neurotrophic factor; *CERS3*: ceramide synthase 3; *CYP3A*: cytochrome P450 family 3 subfamily A member; *CYP2C9*: cytochrome P450 family 2 subfamily C member 9; *CLCA4*: chloride channel accessory 4; *CLCN6*: chloride voltage-gated channel 6; DL-PFC: dorsolateral prefrontal cortex; DMRs: differentially methylated regions; DAGs: DMR-associated genes; *DNMT*: DNA (cytosine-5)-methyltransferase; DMPs: differentially methylated positions; DLPFC-HC: dorsolateral prefrontal cortex hippocampal; *DPPA5*: developmental pluripotency-associated 5; *DDX43*: DEAD-box helicase 43; FS: febrile seizures; *GAD1*: glutamate decarboxylase 1; GAD67: glutamate decarboxylase 67; *GCortR*: glucocorticoid receptor; GFAP: glial fibrillary acidic protein; GCD: granule cell dispersion; HCs: healthy controls; *LY6G5C*: lymphocyte antigen 6 family member G5C; lncRNA: long non-coding RNA; MZ: monozygotic; MTLE: mesial temporal lobe epilepsy; meQTLs: methylation quantitative trait loci; MAPK: mitogen-activated protein kinase; NK: natural killer cell; NeuN: neuronal nuclei; PFC: prefrontal cortex; PB: peripheral blood; PBL: peripheral blood lymphocytes; PMS: polymethylation score; *RELN*: Reelin; *PRDM9*: PR/SET domain 9; *REC8*: meiotic recombination protein; SZ: schizophrenia; *SLC34A2*: solute carrier family 34 member 2; TLE: temporal lobe epilepsy; *TET1*: Tet methylcytosine dioxygenase 1; WB: whole blood; WG1: Watson Grade1.

## Data Availability

Data sharing is not applicable to this article as no datasets were generated or analyzed during the current study.

## Abbreviations

5hmC	5-hydroxymethylcytosine	LTP	Long-term potentiation
5mC	5-methylcytosine	MBD	Methyl-CpG-binding domain
AD	Alzheimer’s disease	mCA	Methylated CA
ADK	Adenosine kinase	mCAC	Methylated CAC
ADO	Adenosine	mCG	Methylated CpG
ADORA2A	Adenosine A2a receptor	MCI	Mild cognitive impairment
AHCY	Adenosylhomocysteinase	Met	Methionine
ALS	Amyotrophic lateral sclerosis	mGluRI	Metabotropic glutamate receptor group I
AMP	Adenosine monophosphate	*mHTT*	Mutant HTT
ASD	Autism spectrum disorders	mQTL	Methylation quantitative trait locus
Aβ	Amyloid beta	MTs	Microtubules
*C9orf72*	Chromosome 9 open reading frame 72	NCI	No cognitive impairment;
D-loop	Displacement loop	NFT	Neurofibrillary tangles
DMRs	Differentially methylated regions	NPCs	Neural progenitor cells
DNAm	DNA methylation	NTD	N-terminal domain
*DNMTs*	DNA methyltransferases	PB	Peripheral blood
DRPs	Dipeptide repeat proteins	PBMCs	Peripheral blood mononuclear cells
FMRP	Fragile X mental retardation protein	PHFs	Paired helical filaments
FTD	Frontotemporal dementia	pHRE	Pathogenic hexanucleotide G4C2 repeats expansion
FXS	Fragile X syndrome	Pol II	RNA polymerase II
G4C2	GGGGCC	p-tau	Phosphorylated-tau
GABA	Gamma-aminobutyric acid	RAN	Repeat-associated non-ATG translation
GoF	Gain of function	RBPs	RNA binding proteins
Hcy	Homocysteine	SAH	S-adenosyl homocysteine
HD	Huntington’s disease	SAM	S-adenosyl methionine
*HTT*	Huntingtin gene	*SNCA*	α-synuclein
ICIs	Intracytoplasmic inclusions	SNP	Single-nucleotide polymorphism
INIs	Intranuclear inclusions	SZ	Schizophrenia
LAS	Lysosomal autophagy system	*TET*	Ten-eleven translocation
lncRNAs	Long non-coding RNAs	TFs	Transcription factors
LoF	Loss of function	TRD	Transcriptional repression domain
LTD	Long-term depression		
